# 3D Time-lapse Imaging and Quantification of Mitochondrial Dynamics

**DOI:** 10.1038/srep43275

**Published:** 2017-02-23

**Authors:** Miguel Sison, Sabyasachi Chakrabortty, Jérôme Extermann, Amir Nahas, Paul James Marchand, Antonio Lopez, Tanja Weil, Theo Lasser

**Affiliations:** 1 Laboratoire d’Optique Biomédicale, Ecole Polytechnique Fédérale de Lausanne (EPFL), Station 17, CH-1015 Lausanne, Switzerland; 2 Department of Organic Chemistry III/Macromolecular Chemistry, Ulm University, Albert-Einstein-Allee 11, 89081 Ulm, Germany; 3 Max-Planck-Institute for Polymer Research, Ackermannweg 10, 55128 Mainz, Germany; 4 Hepia, University of Applied Sciences of Western Switzerland (HES-SO), 4 rue de la Prairie, CH-1202 Genéve, Switzerland

## Abstract

We present a 3D time-lapse imaging method for monitoring mitochondrial dynamics in living HeLa cells based on photothermal optical coherence microscopy and using novel surface functionalization of gold nanoparticles. The biocompatible protein-based biopolymer coating contains multiple functional groups which impart better cellular uptake and mitochondria targeting efficiency. The high stability of the gold nanoparticles allows continuous imaging over an extended time up to 3000 seconds without significant cell damage. By combining temporal autocorrelation analysis with a classical diffusion model, we quantify mitochondrial dynamics and cast these results into 3D maps showing the heterogeneity of diffusion parameters across the whole cell volume.

Mitochondria are organelles present in most eukaryotic cells. They are important for sustaining the energy needs of the host cell via the synthesis of ATP^1^. Besides being the cellular powerhouse, mitochondria are also involved in synaptic transmission and cellular signaling^2^. These fundamental functions translate into continuous mitochondria trafficking, which is an essential characteristic of these organelles, best subsumed by the term “mitochondrial dynamics”^3^. Quantifying these dynamics over a large time span promises to deepen our insight on cellular processes tightly related to neurodegenerative diseases such as Alzheimer’s and Parkinson’s disease^1^,^4^,^5^,^6^.

Confocal fluorescence microscopy remains the most widely used technique for cellular imaging. It provides high spatial resolution and specificity, known from fluorescent markers, making it suitable for intracellular imaging and studying mitochondria morphology. However, available fluorescent markers are prone to photobleaching, such as organic dyes, or are inherently toxic, like quantum dots^7^,^8^, limiting their viability for long-lasting live cell imaging.

Light-sheet micrsocopy is a fast, 3D, and high resolution intracellular imaging technique that also addresses photobleaching. In particular, Planchon, T. A. *et al*.^9^ demonstrated imaging live-cell mitochondrial dynamics using a scanned Bessel beam. Live pig kidney epithelial cells (LLC-PK1 cell line) were imaged over 300 volume stacks displaying a decrease in fluorescence intensity of only <20%. Among these techniques, probably the most advanced is lattice light-sheet microscopy by Chen, B.-C. *et al*.^10^ where imaging mitochondrial dynamics for more than 18 minutes was achieved with limited photobleaching. Undoubtedly, light-sheet microscopy has succeeded in pushing the bounds of intracellular imaging. However, it still relies on fluorescence probes and will eventually be confronted with the same limitations.

The use of gold nanoparticles (AuNPs) as biomarkers for photothermal imaging offers a promising alternative because of their high stability and very low toxicity. However, to accomplish photothermal imaging inside living cells, it is crucial to stabilize the surface of the AuNPs with water-soluble, biocompatible ligands that can withstand various physiological conditions. Stability of the AuNPs at varying pH and in the presence of proteases located in vesicles during endosomal uptake processes or in the reductive environment in the cytoplasm are essential to provide suitable intracellular markers for time lapse studies^11^. Protein based polymeric surface coatings offer the additional advantage as multiple functionalities could be incorporated at the level of the polymer. This ensures characterization by standard polymer analytics and guarantees the presence of all required functions at the particle surface after coating^12^. In this way, photothermal imaging AuNPs could be envisaged carrying the desired functionalities for targeting sub-cellular organelles, such as mitochondria, inside living cells.

The photothermal contrast mechanism relies on a temporally modulated refractive index in the near vicinity of the AuNPs resulting from heat dissipated by the AuNPs’ plasmon-enhanced absorption. This contrast mechanism is the basis for photothermal optical lock-in optical coherence microscopy (poli-OCM), which provides two distinct imaging modalities: a dark-field mode (dfOCM) for imaging the 3D cell volume, and a poli-mode, utilizing functionalized AuNPs for highly specific 3D mitochondria imaging^13^.

In this work, we report the synthesis of a novel and biocompatible protein-based biopolymer for surface functionalization of AuNPs. The biopolymer is comprised of multiple functional groups imparting enhanced cellular uptake and mitochondria targeting. Using these AuNPs, 3D mitochondria specific poli-OCM imaging during 3000 seconds was demonstrated without any loss of contrast. Finally, we quantified mitochondrial dynamics, per voxel, using temporal autocorrelation analysis based on a classical diffusion model, which allowed us to extract mitochondria diffusion time *τ*_*D*_ and other diffusion parameters. The novelty of our method resulted in cells segmented into sub-volumes providing 3D parameter maps.

## Results and Discussion

### Synthesis of mitochondria specific AuNPs

**Figure 1:**
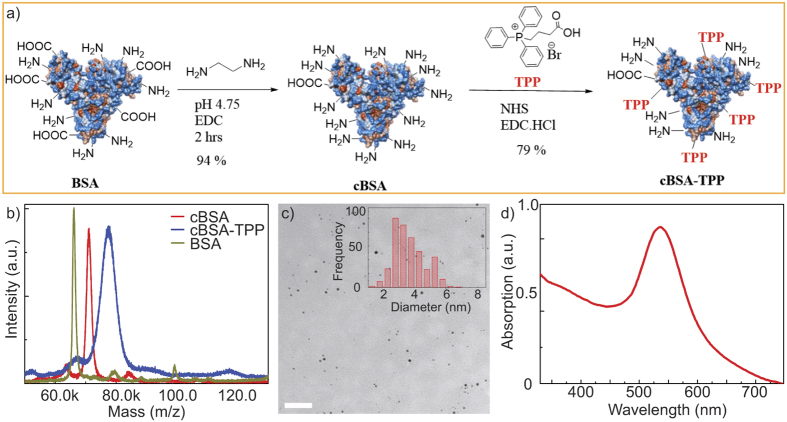
(**a**) Synthesis scheme of globular cationized protein (cBSA-TPP) with multiple mitochondria targeting TPP groups attached. (**b**) MALDI-ToF spectra (matrix: sinapinic acid) indicate successful functionalization from the progressive increase in molecular weight from BSA (calculated 66.00 kDa, measured 66.13 kDa), cBSA (calculated 71.05 kDa, measured 71.04 kDa) and cBSA-TPP (calculated 77.62 kDa, measured 77.51 kDa). Approximately 19 TPP units were attached. (**c**) Low resolution transmission electron microscopy (TEM) image of cBSA-TPP coated AuNPs. Scalebar: 50 nm. Inset: Size distribution histogram indicating average diameters of the AuNPs of 3.7 ± 0.9 nm. (**d**) Characteristic absorption spectra of the as-synthesized AuNPs highlighting the surface plasmon peak centered at 536 nm.

We synthesized mitochondria targeting AuNP biomarkers tailor-made for poli-OCM imaging. The synthesis, functionalization, and characterization of these AuNPs are summarized in Fig. 1a–d. The blood plasma protein is known to be biocompatible and provides many reactive carboxylic acid and amino groups that can be further modified. First, all accessible carboxylic acid groups were converted into primary amino groups by applying ethylenediamine and the coupling reagent EDC according to a literature-known procedure^14^. After dialysis, globular polycationic bovine serum albumin (cBSA) was obtained having the ability to interact with cellular membranes, facilitate cellular uptake, and cytosolic release by Clathrin-mediated endocytosis^15^. To accomplish mitochondria targeting, lipophilic triphenyl phosphonium groups (TPP) were attached to cBSA^16^. According to the MALDI-ToF mass spectra (Fig. 1b), about 19 TPP groups were attached to the cBSA surface yielding cBSA-TPP in good yields. cBSA-TPP was then purified and subjected to AuNP preparation. We used a strong reducing agent, NaBH_4_, to reduce Au-salt (HAuCl_4_) in the presence of cBSA-TPP and yielded monodispersed spherical AuNPs with diameters centered around 3.7 ± 0.9 nm (Fig. 1c). The inset histogram map confirms the relative narrow size distribution and Fig. 1d shows the characteristic plasmonic peak of the prepared AuNPs. Since the stability of the cBSA-TPP passivated AuNPs in biological media is crucial for cellular uptake and subsequent observation of their movement without prior aggregation, dynamic light scattering experiments were performed in DMEM medium (see Fig. S2b). No indication of severe aggregation, as little increase of the particle size, was observed, which is believed to be due to the alteration of ionic strength in biological medium as compared to its aqueous counterpart^17^.

**Figure 2:**
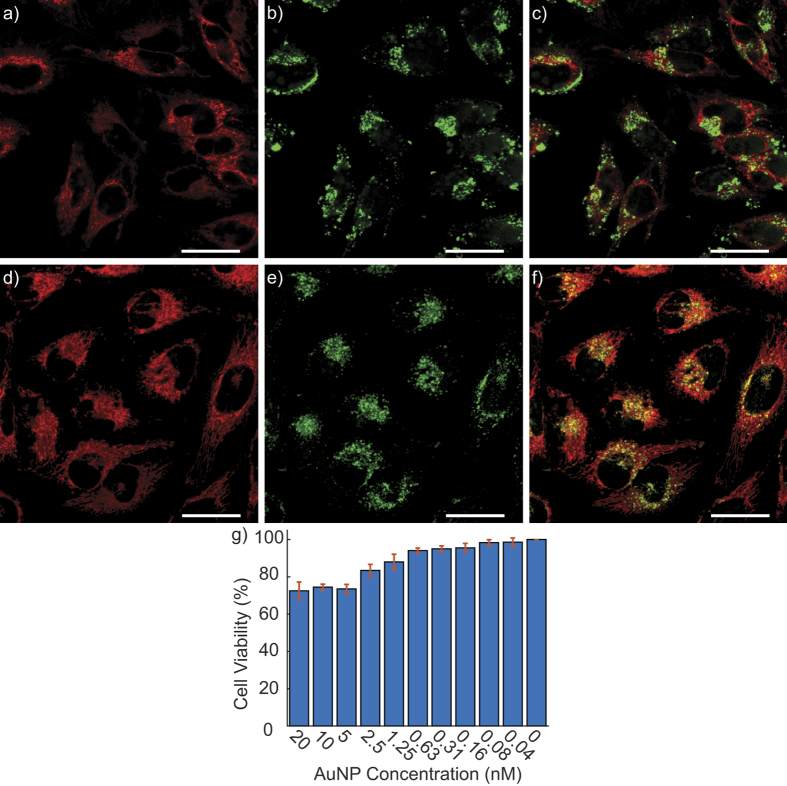
Fluorescence confocal images of HeLa cells incubated with AuNP-cBSA (**a**–**c**) and AuNP-cBSA-TPP (**d**–**f**) co-stained with MitoTracker. (**a** and **d**) are the MitoTracker images whereas (**b** and **e**) are the FITC tagged AuNPs. Their overlays in (**c** and **f**) have Pearson’s coefficients of 0.29 and 0.69 for AuNP-noTPP and AuNP-TPP respectively. Scalebars: 30 *μ*m. (**g**) Cell viability test showing 95% viability HeLa cells incubated with 0.31 nM of mitochondria targeting AuNPs.

In order to demonstrate the specificity of our AuNP biomarkers, HeLa cells with AuNP labeled mitochondria were co-stained with MitoTracker Red, a standard mitochondria specific dye. These AuNPs were linked with FITC fluorophores which enabled imaging via confocal laser scanning microscopy and colocalization with MitoTracker staining. We conducted this test using two different kinds of AuNP functionalization; the first group of AuNP-cBSA was attached with TPP (AuNP-cBSA-TPP) while the second one was not (AuNP-cBSA). Figure 2a–f shows the fluorescence confocal images of the HeLa cells with MitoTracker (red), AuNP-FITC (green), and their overlays. From these images, we observed good colocalization of AuNP-cBSA-TPP with MitoTracker giving a Pearson’s coefficient of 0.69 (Fig. 2d–f) as opposed to 0.29 for AuNP-cBSA (Fig. 2a–c). These results are indicative of the specificity of our AuNP biomarkers and substantiate their use for mitochondria specific poli-OCM imaging. In addition, we also investigated the biocompatibility of our AuNP labels through a cell viability assay (Fig. 2g and Fig. S3). We obtain 95% cell viability at 0.31 nM AuNP concentration which is the typical AuNP labeling concentration we use for poli-OCM.

### Quantifying mitochondrial dynamics

We quantified mitochondrial dynamics by exploiting fast and specific 3D imaging via poli-OCM and using a diffusion model considering the mitochondria as freely diffusing particles inside the voxel volume. Knowing the high complexity of the intracellular structure, we argue that using a voxel sized sampling volume allows the use of a diffusion model supposing a homogeneous environment. This model permits to assess the general mitochondrial dynamic and to cast our results in a parameter space known and used in correlation microscopy.

Our correlation analysis closely follows the principles of image correlation spectroscopy (ICS) developed by Petersen *et al*.^18^ and Wiseman *et al*.^19^,^20^ that extended fluorescence correlation spectroscopy (FCS) to full 2D imaging methods. ICS based correlation analysis on pixel-wise intensity fluctuations provide the diffusion time of the particles under investigation. Merging photothermal detection and correlation spectroscopy was previously introduced^21^,^22^ but not with 3D imaging or extended time scales (up to 1 hour)^23^. In a similar manner, phase correlation imaging has also been demonstrated as a robust technique for studying cell dynamics^24^ even without the use of specific labels. As we have previously stated, using the poli-OCM we achieved fast 3D mitochondria specific live cell imaging up to 3000 seconds without any loss of contrast (see Fig. S6 and Movie S1,S2,S3).

Stepping from 2D to 3D imaging, the voxel-wise autocorrelation function can be stated as


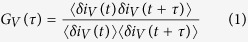


where the indices V point to the individual voxels i.e. the sampling volumes, 〈〉 denotes time averaging, *i*_*V*_(*t*) the time-dependent intensity and *τ* the temporal lag. The temporal intensity fluctuation is then defined as *δi*_*V*_(*t*) = *i*_*V*_(*t*) − 〈*δi*_*V*_(*t* + *τ*)〉. Assuming a 3D Gaussian sampling volume, (see Fig. S5), the processed intensity data are fitted with the model for classical 3D diffusion behavior


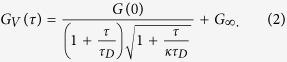


Here 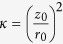 where *r*_0_ and *z*_0_ represent the lateral and axial extent of the sampling volume, *τ*_*D*_ is the diffusion time, and *G*_∞_ = lim_*τ*_ _→_ _∞_*G*_*V*_(*τ*)^20^,^25^. In FCS,  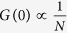, where *N* is the mean number of diffusing particles. *G*(0), in turn, is dependent on the signal to noise ratio and also considers “independent particles”^26^, which in the case of poli-OCM can be influenced by AuNP labelling efficiency and interparticle coupling^27^; an in depth analysis of this dependence is beyond the scope of this study. All instrument parameters, *r*_0_ and *z*_0_, were evaluated by measuring the point spread function (PSF), fitting it with a Gaussian profile, (Fig. S5) and by taking into account the coherence length which defines the axial extent of the PSF^28^,^29^. For a more in depth discussion of coherent correlation analysis please refer to previous papers in OCCS^28^,^29^. Due to the interferometric detection, our signal has extremely high sensitivity to phase and not only intensity in contrast to ICS of Wiseman and Petersen.

**Figure 3:**
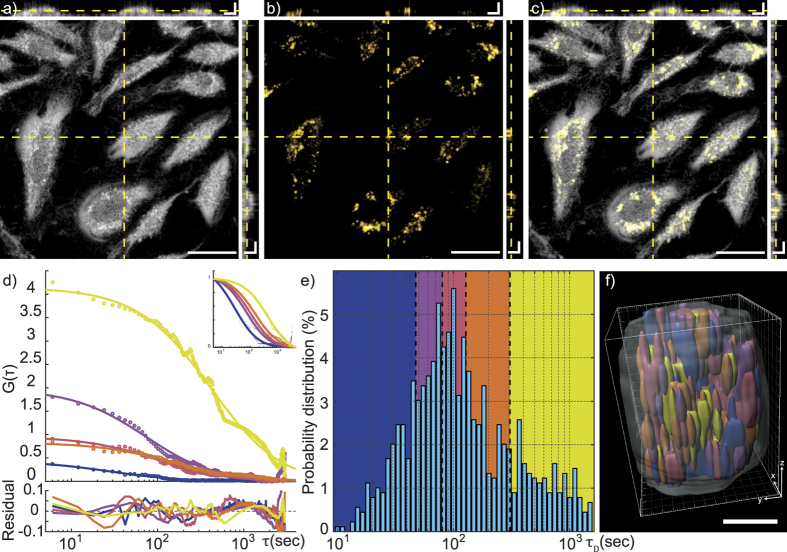
En face and orthogonal slices of (**a**) 3D dark-field OCM and (**b**) poli-OCM tomograms and (**c**) their overlay. In (**d**) we show selected autocorrelation data with corresponding fits and their residuals. Inset: normalized autocorrelation and fits. (**e**) Probability distribution of all extracted diffusion times from each sub-volumes in the entire cell is shown. The color-coded segments of (**e**) each represents approximately 20% of the complete cell volume. (**f**) 3D rendering (diffusion time map) using data from (**e**) illustrating the high heterogeneity of mitochondrial dynamics within the cell. The semi-transparent grey volume outlines the entire cell volume. (**d**–**f**) Shares the same color-code meaning each curve in (**d**) comes from the same colored segment in e) as well as the same colored sub-volume in (**f**) Scalebars: (**a**–**c**) 30 *μ*m en face and 7.5 *μ*m orthogonal slices, (**f**) 5 *μ*m.

The dual imaging modality of the poli-OCM is exhibited in Fig. 3a–d where we show en face and orthogonal slices of the 3D dfOCM (Fig. 3a), photothermal OCM (Fig. 3b) tomograms, and their overlay (Fig. 3c). The orthogonal slices emphasize the 3D specific imaging and localization we achieve using the poli-OCM; we are capable of imaging the entire cell morphology (Fig. 3a) as well as only the AuNP labeled mitochondria (Fig. 3b). For the purposes of our temporal autocorrelation analysis we image individual cells in a scan range of 25 *μ*m × 25 *μ*m. Alternating between dfOCM and poli-mode, tomograms similar to (Fig. 3a and b) were acquired to monitor both the motion of the entire cell (dfOCM) and mitochondrial dynamics (photothermal OCM).

The temporal autocorrelation was calculated for each voxel in the 3D poli-OCM tomogram time series following equation (1). The data was then segmented into sub-volumes of 2 × 2 × 2 voxel (0.58 *μ*m × 0.58 *μ*m × 2.05 *μ*m) where the autocorrelation was averaged and subsequently fitted with the diffusion model given by equation (2) using a non-linear least squares solver. Our method allowed the extraction of diffusion parameters from each of these segmented sub-volumes resulting in a 3D diffusion time map (Fig. 3f). Furthermore, the 2 × 2 × 2 voxels sub-volume we use are related to the measured dimensions of our point spread function (PSF).

The data shown in Fig. 3d represent five sub-volumes with different diffusion times (see matched color code of histogram in Fig. 3e) underlining the high variation of the mitochondrial dynamics across the full cell volume. The very small residuals between the autocorrelation and the model indicate a good fit and give confidence to the validity of the model. Additionally, the normalized autocorrelations (Fig. 3d, inset) demonstrate even more the large variation of *τ*_*D*_. The probability distribution (*pdf*) of the *τ*_*D*_ extracted from all sub-volumes in the entire cell (Fig. 3e) demonstrates a spread of over two orders of magnitude with a *τ*_*D*_ ranging from 10 to 1.5 × 10^3^ seconds having a mean value of 214 sec. (median value of 101 sec.). We divided the *pdf (τ*_*D*_) into five color-coded intervals, each representing 20% of the total cell volume (Fig. 3e). These intervals were used to render a 3D map of the diffusion time, as shown in Fig. 3f and Fig. S9 with each sub-volume color-coded according to the *pdf (τ*_*D*_). This 3D map allows us to determine and locate regions within the cell featuring high or low mitochondrial dynamics. These extracted *τ*_*D*_ can be further related, by the Stokes-Einstein relation (see Fig. S8), to the viscosity values inside our sampling volumes. Assuming a mitochondria hydrodynamic radius of 500 nm we deduced viscosity values ranging from 0.18 to 26.7 (Pa⋅s), which are still within the range predicted by the model of Kalwarczyk *et al*.^30^. In addition, the associated diffusion constants we calculate from these data coincide with published quantified cellular dynamics using phase correlation imaging^24^.

In addition to the high heterogeneity of mitochondrial dynamics, regions within the cell, where it was not possible to extract any diffusion parameters were also observed. As elaborated in Section 6 of the Supporting Information, this region exhibits a high overlap with the locations in the corresponding dfOCM (dark-field OCM) time series showing lower scattering signal. We associate this low scattering regions to the nucleus of the cell where no mitochondria are expected.

The high heterogeneity of diffusion parameters over the cell volume is an observed phenomenon resulting from our data and analysis. This is not surprising given the known complexity of the intracellular environment^31^,^32^,^33^, especially considering the size of mitochondria and their tendency to form networks. Furthermore, the works of Planchon *et al*.^9^ and Chen, B.-C. *et al*.^10^ also observed this variety in mitochondrial dynamics using a completely different and independent imaging concept. We would like to reiterate that, unlike fluorescence, poli-OCM imaging is not prone to photobleaching at all; as shown in Fig. S6, there is no decrease in signal contrast even after acquiring 500 poli-OCM tomograms. We, nevertheless, consider lattice light-sheet microscopy as the golden standard for imaging live intracellular dynamics and poli-OCM imaging compares well with these published time-lapse acquisition protocols. These previous works in light sheet microscopy^9^,^10^ demonstrated similar high heterogeneity of mitochondrial dynamics and can therefore be considered as an independent validation of our observations.

## Conclusion

In this paper, we report highly specific fast 3D imaging of mitochondrial dynamics inside living HeLa cells. We prepared narrowly dispersed AuNPs with a protein coating based on the plasma protein BSA, functionalized with positive ammonium and multiple TPP groups. The positive ammonium groups improve cell membrane attachment and endocytosis while the TPP groups provide intracellular mitochondria targeting. All AuNPs used in this study were decorated by the functionalized protein coating approach. The photostability and limited toxicity of these markers enabled true long-lasting time lapse live cell imaging. Finally, we used these functionalized AuNPs for fast 3D poli-OCM imaging to quantify mitochondrial dynamics via temporal autocorrelation analysis resulting to 3D diffusion parameters maps. We believe that this method provides a novel perspective of mitochondrial dynamics and paves an innovative way for investigating its relation to various not yet understood metabolic diseases.

## Methods

### Preparation of functionalized protein coated AuNPs

#### Materials used

Cationized bovine serum albumin (cBSA) was synthesized following the procedure reported previously by our group^34^ N-hydroxy-succinimide (NHS, 99%), (3-Carboxypropyl) triphenylphosphonium bromide (TPP), 98% and N-(3-Dimethylaminopropyl)-N′-ethylcarbodiimide hydrochloride (EDC, 98%) were procured from Sigma-Aldrich. Hydrogen tetrachloro aurate (III) trihydrate, ACS, 99.99% was obtained from Alfa Aesar. Sodium borohydride 98% was bought from Acros Organics. All chemicals were used as received without further purification. Vivaspin ultrafiltration tubes were purchased from GE healthcare. Ultra-pure milli-Q water was used for all experiments involving water.

#### Synthesis of cBSA-TPP

TPP (80 mg), NHS (30 mg) and EDC.HCl (40 mg) were dissolved in 0.5 mL of degassed dimethylformamide (DMF) solution. This mixture was stirred at room temperature under argon atmosphere for overnight. Next day, cBSA (20 mg) dissolved in 20 mL milli-Q water was added and reacted overnight at room temperature. The product was washed through vivaspin 20 (MWCO 30 K) ultracentrifuge tube to separate the unreacted reactants. Finally, the solution was kept at 4 °C for future use. Figure S1 shows the zeta potential and XPS data of the conjugate.

#### Synthesis of cBSA-TPP coated AuNPs

AuNPs with desired sizes were synthesized and functionalized for mitochondria labeling. Briefly, aqueous HAuCl_4_ solution (60 *μ*L, 10 mM) and cBSA-TPP (globular cationic bovine serum albumin with triphenyl phosphonium cations, 50 *μ*L, 10 mg/mL) were mixed and the solution was diluted to 250 *μ*L with milli-Q water. Freshly prepared NaBH_4_ solution (6 *μ*L, 3 mg/mL) in chilled milli-Q water was added rapidly to the solution which yielded monodispersed, spherical AuNPs with diameter of approximately 4 nm. Then, the reaction mixture was stirred for 30 minutes and washed with water to remove excess of NaBH_4_. By varying the amount of added NaBH_4_, various sizes of the AuNPs were obtained. In particular, the AuNPs used in the poli-OCM imaging experiments had diameters of 3.7 ± 0.9 nm. Corresponding XPS peaks of AuNPs are presented in Fig. S2a.

#### AuNP characterization

Plasmonic absorption was recorded using TECAN infinite M1000 microplate reader. Zeta-potential and DLS measurements were performed using a Malvern Zetasizer ZEN3600 (Malvern Ltd, Malvern, UK) at 20. A JEOL 1400 transmission electron microscope was used to obtain bright field TEM images of the AuNPs. X-ray photoelectron spectroscopy (XPS) data were recorded on a Physical Electronics PHI 5800 ESCA System using mono-chromatized Al K*α* radiation (13 kV, 250 W).

### Cell viability assay

HeLa cells were cultured in Dulbecco’s Modified Eagle Medium (DMEM, Gibco) with 10% fetal bovine serum, 1% penicillin/streptomycin with phenol-red and seeded at 6,500 cells/well in a white 96-well (half-area) plate. The cells were left to adhere overnight at 37 °C and 5% CO_2_ and afterwards different concentrations of AuNPs were added into each well. The treated cells were subsequently incubated with the AuNPs for approximately 30 hours at 37 °C, 5% CO_2_. After incubation, the cells were washed with phosphate buffer to remove the excess AuNPs present in the medium and re-incubated with DMEM medium. The cells were further incubated for 4 hours before the addition of Tox-8 reagent with phenol red free DMEM medium. After 2 hours incubation with Tox-8, the emission intensity was measured by a Tecan Infinite M1000 microplate reader (*λ*_*ex*_ = 570 nm, *λ*_*em*_ = 590 nm). We used wells without cells but with Tox-8 reagent as controls. Each experiment was performed in quadruplicates. The cell viability (*V*) was calculated according to the following equation


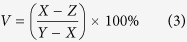


where *X* is the average emission with the treatment of AuNPs at various concentrations, *Y* is the average emission of the experimental groups without the treatment of the AuNPs, and *Z* is the average emission of the culture medium background.

### Confocal microscopy

HeLa cells were incubated in Dulbecco’s Modified Eagle Medium (DMEM, Gibco) with 10% fetal calf serum (FCS, Gibco) and non-essential amino acids in a humidified incubator at 37 °C and 5% CO_2_. For confocal laser scanning microscopy (CLSM), cells were seeded onto an Ibidi *μ*-slide 8-well and incubated with FITC labelled protein coated AuNPs for 24 hours. We used two different kinds of protein coating: one with TPP attached and the other without TPP attached in its backbone. Fluorescence confocal laser scanning microscopy (CLSM) images were acquired with a 63 × 1.4 NA oil immersion plan apochromatic LSM 710, Axio Observer objective. Fluorescence of FITC was excited by a HeNe laser *λ*_*ex*_ = 488 nm (AOTF transmission set to 10–20%) and was set to emission maximum at 525 nm. We added 1 *μ*M MitoTracker Red in each well-plate and excited at 560 nm with an emission maximum at 625 nm.

### poli-OCM imaging

**Figure 4:**
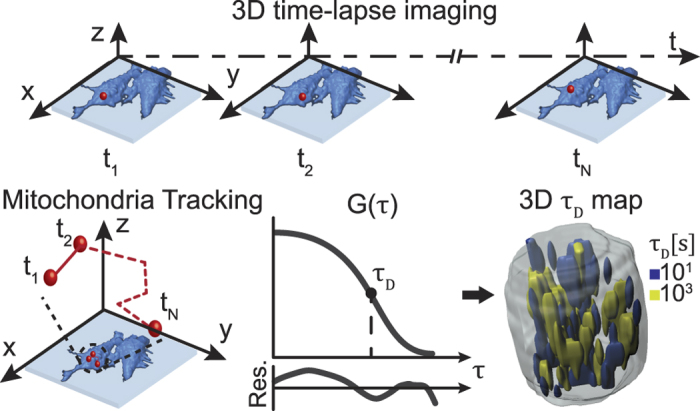
Schematic summarizing step by step imaging and data extraction for mitochondrial dynamics. 3D time lapse imaging of live HeLa cells was performed using the poli-OCM localizing the AuNP labeled mitochondria. Temporal autocorrelation analysis was applied over the whole cell volume resulting in a 3D map of mitochondria diffusion time. In addition, imaging with the poli-OCM also enables mitochondrial dynamics quantification via tracking individual AuNP-labeled mitochondria but is outside the scope of this work.

The 3D time-lapse imaging and analysis we implemented for the quantitative assessment of mitochondrial dynamics is summarized schematically in Fig. 4.

#### Cell culture and AuNP labelling

HeLa cells were seeded and incubated overnight onto an Ibidi *μ*-slide 8-well with 250 *μ*L of cell culture medium (Dulbecco’s Modified Eagle Medium (DMEM, Gibco), 10% fetal bovine serum, and 1% penicillin and streptomycin antibiotics). 2 *μ*L of 40 nM AuNP solution were then added into the media and again incubated overnight. Finally, the cells were washed with PBS and fresh culture medium was added before imaging. During poli-OCM imaging, the Ibidi *μ*-slide 8-well was housed in a custom built micro-incubator, where the temperature was regulated at 37 °C and humidified premixed (5% CO_2_) air was pumped continuously.

#### poli-OCM Instrumentation

The specifics of the OCM instrument as shown in Fig. S4 have been previously described and characterized elsewhere (xfOCM^35^, dfOCM^36^ and poli-OCM^13^). A broadband laser source centered at 800 nm (Δ*λ* = 135 nm) is used as a source for optical coherence imaging. An axicon shapes the illumination beam into a Bessel beam which propagates across the scanning unit and is focused on the sample by a high-NA objective (25×, NA = 0.8, Carl Zeiss). The backscattered field is collected by the same objective and superimposed with the reference beam; the resulting interference is finally recorded using a custom-made spectrometer. The Bessel beam configuration of our poli-OCM ensures an extended depth of field or a uniform lateral resolution over the full 3D cellular volume. The poli-OCM, as used in this study, provides a lateral and axial resolution of 0.53 *μ*m and 2.15 *μ*m respectively over an extended depth of focus of 50 *μ*m. The experimental measurement of the resolution is discussed in the supporting information. The photothermal contrast of the poli-OCM^13^ is achieved by exciting the plasmonic resonance of the AuNPs using a 532 nm solid-state laser. This photothermal heating beam is intensity modulated at 150 kHz using an AOM before being coupled into the OCM scanning unit by a dichroic mirror (D1; 720DCXR, Chroma Technology). This intensity modulation induces a similarly modulated index of refraction around the AuNP which in turn generates a modulated backscattered signal. This backscattered field is superimposed with a phase modulated reference field (150 kHz) enabling heterodyne detection of the sinusoidally modulated signal. In accordance with C. Pache *et al*. we operate at an A-scan rate of 3900 Hz with an integration time of 250 *μ*s^13^. At this operating parameters the temperature increase in the vicinity of an individual AuNP is approximately 1 K^13^,^37^. During imaging, we used approximately 6 mW for OCM probe beam and 3 mW average power for the photothermal heating laser.

#### Time lapse imaging

Aiming for the extraction of diffusion parameters of the AuNP labeled mitochondria requires fast 3D imaging with short time intervals while extending the experiment over a prolonged acquisition period (100 to 10^3^ seconds). A total of 1000 3D tomograms were acquired over a volume of 25 × 25 × 50 *μ*m^3^ (approximately 0.29 × 0.29 × 1.028 *μ*m^3^ per voxel) while alternating between dfOCM and poli-OCM. This resulted in two time series each having 500 3D tomograms and a time interval of 6 seconds between tomograms. This scan protocol provided us with sufficient sampling necessary for our autocorrelation analysis. Similarly, poli-OCM imaging allows for 3D tracking of AuNP labeled mitochondria but this is not within the scope of this current work.

## Additional Information

**How to cite this article:** Sison, M. *et al*. 3D Time-lapse Imaging and Quantification of Mitochondria Dynamics. *Sci. Rep.* **7**, 43275; doi: 10.1038/srep43275 (2017).

**Publisher's note:** Springer Nature remains neutral with regard to jurisdictional claims in published maps and institutional affiliations.

## Supplementary Material

Supporting Video 1

Supporting Video 2

Supporting Video 3

Supplementary Information
