# Supplementary material for: 3D Time-lapse Imaging and Quantification of Mitochondrial Dynamics
Source: Sci Rep. 2017 Feb 23;7:43275. doi: 10.1038/srep43275 (PMC5322395; doi:10.1038/srep43275)
Supplement: Supplementary Information [file srep43275-s4.pdf]

Supporting Information

Title: 3D Time-lapse Imaging and Quantification of Mitochondrial Dynamics

Miguel Sison, [1, †, *] *Sabyasachi Chakrabortty,*[2,3, †] *Jérôme Extermann,* [1,4] *Amir Nahas,* [1] *Paul James Marchand,* [1] *Antonio Lopez,* [1] *Tanja Weil,* [2,3] *and Theo Lasser* [1,*]

S1. Functionalized protein coated Au nanoparticle preparation

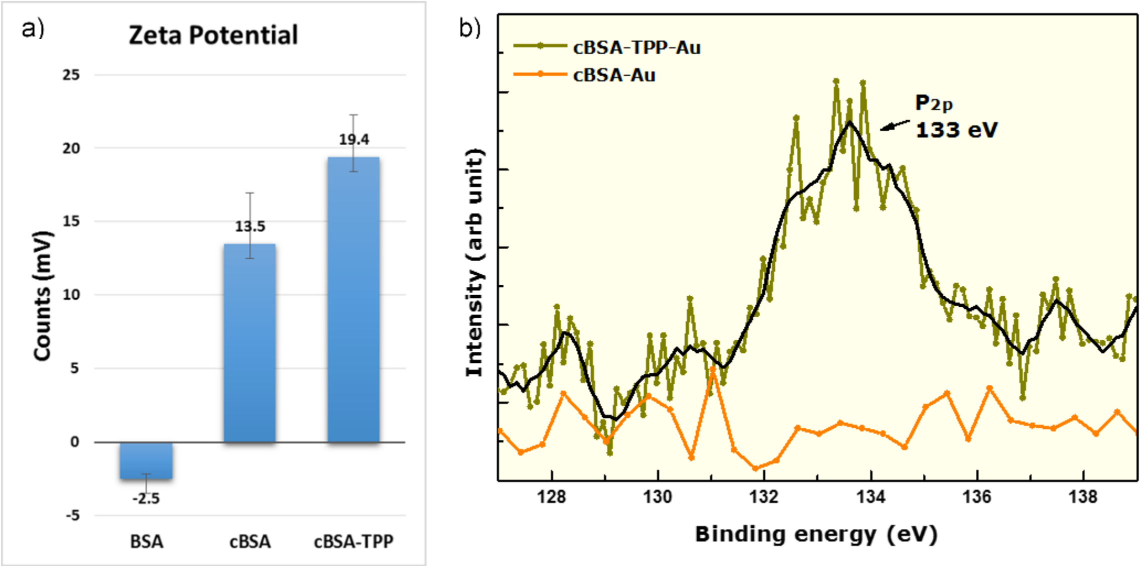

Figure S1. a) Zeta-potential values of BSA, cBSA, and cBSA -TPP conjugates. Increase in zeta-potentials proceeds in line with the expected increase in the number of positive charges after each reaction step. b) XPS spectra of the protein backbone before and after TPP conjugation, where the respective peaks of P_{2p} is clearly observed.

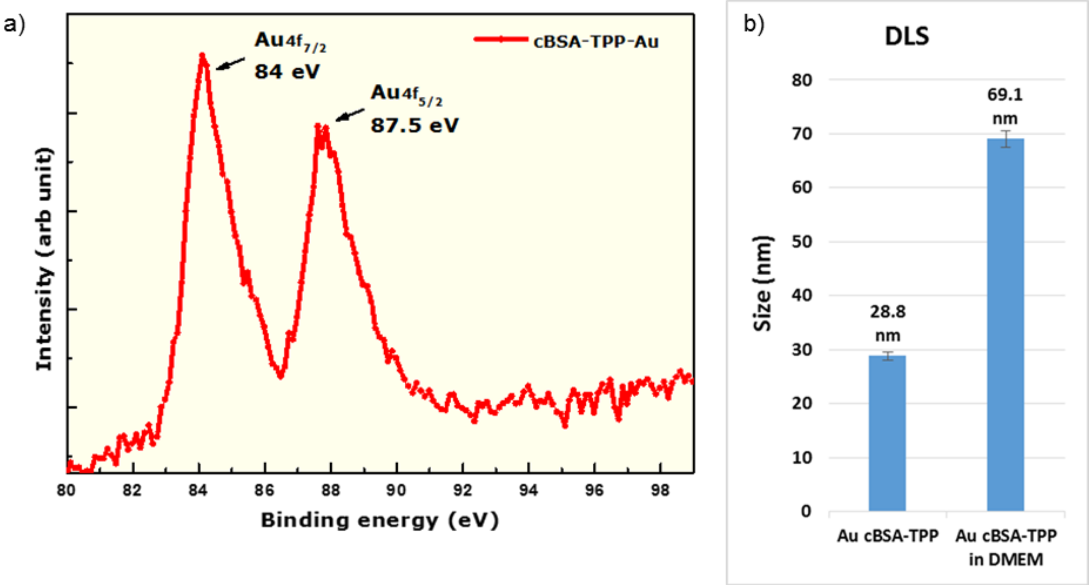

Figure S2. a) XPS spectra of cBSA-TPP passivated Au NPs, where the respective peaks of $Au_{4f7/2}$ and $Au_{4f5/2}$ are readily seen. b) Dynamic Light Scattering (DLS) data of as-synthesized Au NPs in water and DMEM medium. No indication of aggregation is observed.

S2. Cell viability test

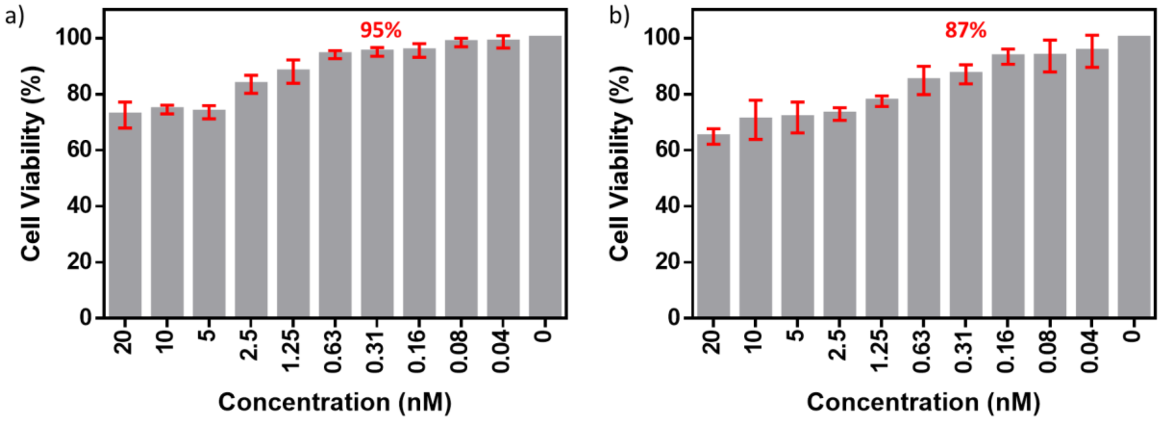

Figure S3. Cell viability data of cBSA-TPP coated Au NPs with (a) 30 h incubation with no light irradiation, (b) 30 h total incubation and after 24 h 532 nm LED light irradiation at ~ 5 mW power with continuous illumination for 5 min. For obtaining poli-OCM image in this work, we used 0.31nM AuNP which clearly stayed in the window where most of the cells were alive (95% and 87% respectively).

In addition to the cell viability test included in the main text, we investigated the viability of Hela cells with AuNP labeled mitochondria exposed to 532nm illumination. A second 96-well plate was prepared following the same procedure as in the experimental section and incubated with the same concentration of AuNPs. This plate was illuminated with light from a 532nm LED array with approximately 5mW of power for 5 minutes continuously. Exposure to the 532nm light was done after washing the excess AuNPs from cell culture. As seen in Figure S3b we still achieved good cell viability at 87% even with this light exposure.

The photothermal contrast of our poli-OCM is achieved by scanning a focused intensity modulated 532nm beam with an average power of 2mW. Using an integration time of 250µs for each A-Scan location (1 pixel along the lateral scan) this translate to 0.5µJ of light energy distributed over the extended focal depth. Please be aware that for OCM we only need to do 2D scan to achieve 3D tomograms because a single A-Scan records the full axial profile. The induced light stress is therefore negligible compared to fluorescence confocal microscopy. Even with 5mW exposure for 5 minutes (1.5J equivalent to 3x106 times more energy) we still achieve 87% cell viability; however, this is through wide-field illumination over the whole cell culture and is not directly comparable to our imaging conditions.

S3. poli-OCM schematic and system parameters

A confocal fluorescence channel was also added to the poli-OCM using a dichroic mirror (D2; TL600, Chroma Technology). A 632nm HeNe laser is used as an excitation source spectrally separated from the fluorescence detection path by a dichroic mirror (D3; z647rdc, Chroma technology). This fluorescence signal is filtered by a bandpass filter (HQ680/35, Chroma technology) and detected by an avalanche photodiode (SPCM-AQR-14-FC, PerkinElmer). The addition of the confocal channel enables simultaneous dfOCM and fluorescence imaging.

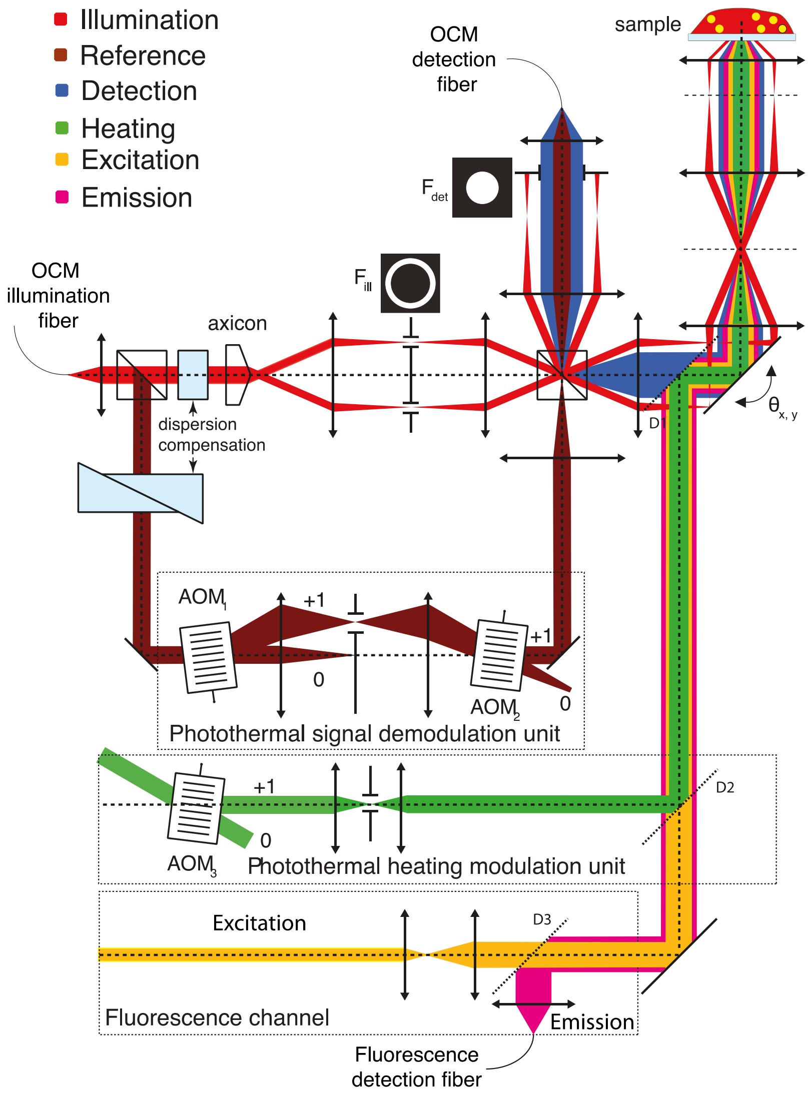

Figure S4: Photothermal optical lock-in optical coherence microscopy setup with an added fluorescence channel.

S3.1. Summary of system parameters

S3.1.1 OCM Imaging system

- Central wavelength: 800nm
- Bandwidth: 135nm
- Effective NA: 0.68
- A-Scan rate: 3900Hz
- Integration time: 250µs
- Power: 6mW

S3.1.2 Photothermal excitation

- Wavelength: 532nm
- Modulation frequency: 150kHz
- Average power: 3mW

S3.1.3 Fluorescence imaging

- Wavelength: 632nm
- CW Power: 80µW

S3.2. Scanning protocol

- The OCM images shown in Figure 3 were acquired by scanning 512×512 pixels covering an area of 150×150 µm^2 along the *x*-*y* plane.
- The 3D time lapse tomograms used for the autocorrelation analysis (Figure3) were acquired by scanning 86×86 pixels covering 25×25 µm^2 along the *x*-*y* plane.
- The spectrometer used for OCM allows imaging an axial extent of 700µm in air (≈526µm in water with 1.33 index of refraction) over 512 pixels.
- For all our OCM imaging we maintain a point spread function oversampling i.e. a voxel sampling of approximately 0.29µm/voxel laterally 1.028 µm/voxel axially.

S3.3. PSF measurement

We measure the point spread function (PSF) of the poli-OCM experimentally by imaging 50nm AuNPs suspended in PDMS (n = 1.4) as shown in Figure S5. We extract the linear profiles laterally and axially along the dashed lines in Figure S5 and fit a Gaussian following

$$PSF(s) = A\exp\left(-\frac{(s-\mu)^2}{2\sigma^2}\right) \quad \text{(S1)}$$

where s represents the spatial dimension (x, y or z). Table S1 lists the parameters of the fitted Gaussian laterally (x,y) and axially (z). From this fit we derive the lateral and axial extent, r_0 and z_0, used in our classical diffusion model which corresponds to the e^{-2} radius of the PSF along both dimensions.

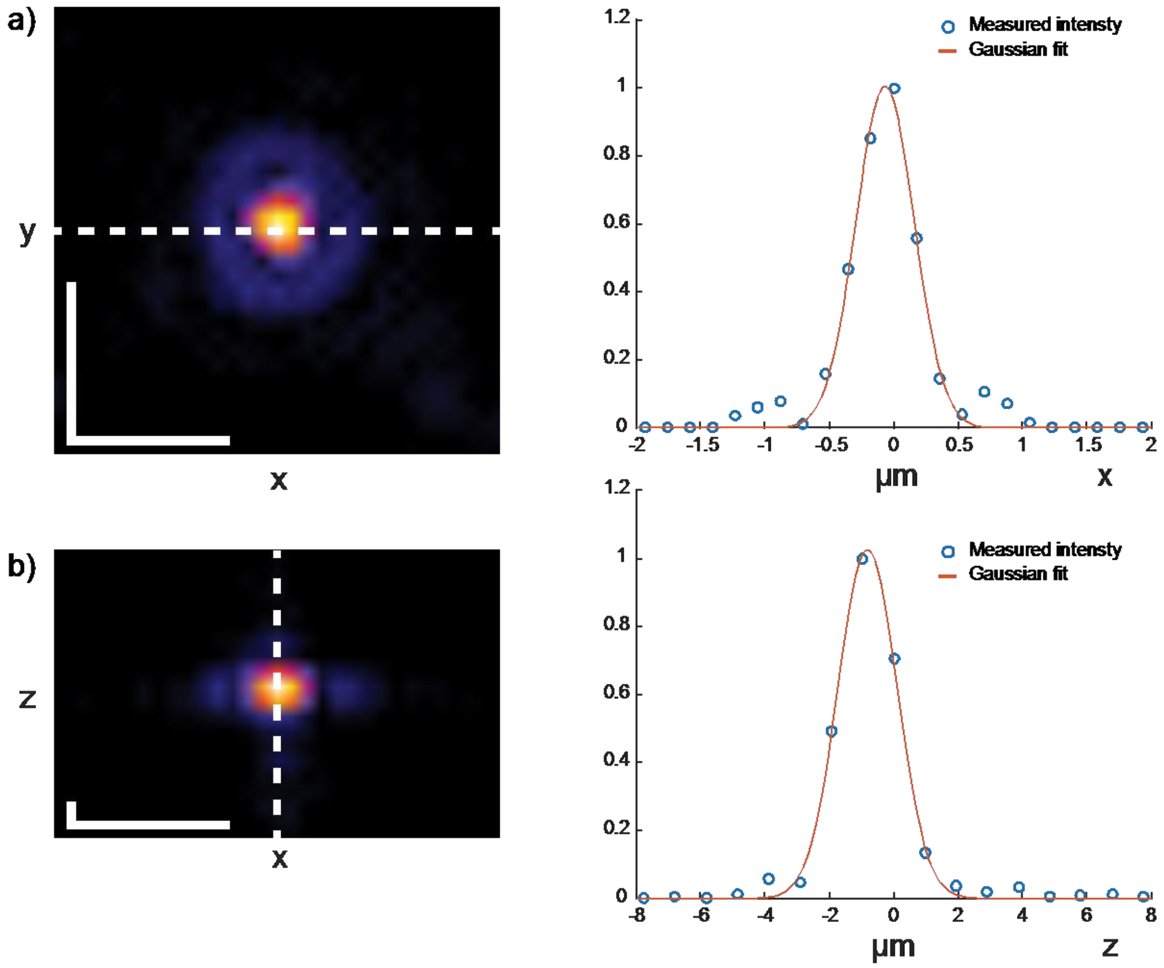

Figure S5: a) En face and b) orthogonal slice of a poli-OCM image of a 50nm AuNP in PDMS and their normalized profile taken along the dashed line with a corresponding Gaussian fit. Scalebar: 2μm.

Table S1. Gaussian fit and PSF parameters of the poli-OCM

	Lateral	Axial
A	1.004	1.026
μ	-0.067 µm	-0.811 µm
σ	0.226 µm	0.913 µm
FWHM	0.532 µm	2.150 µm
$r_{e^{-2}}$	r_0= 0.320 µm	z_0= 1.291 µm

S3.4. Contrast stability of live cell poli-OCM imaging

We demonstrate the stability of the photothermal contrast of our poli-OCM by monitoring the total signal intensity for each of the 3D tomograms over the entire time series. Unlike most fluorescent dyes, we expect stable poli-OCM signal even with prolonged continuous imaging. Figure S6 shows the behavior of the poli-OCM signal over a span of 3000 seconds, which clearly shows no decrease in signal intensity.

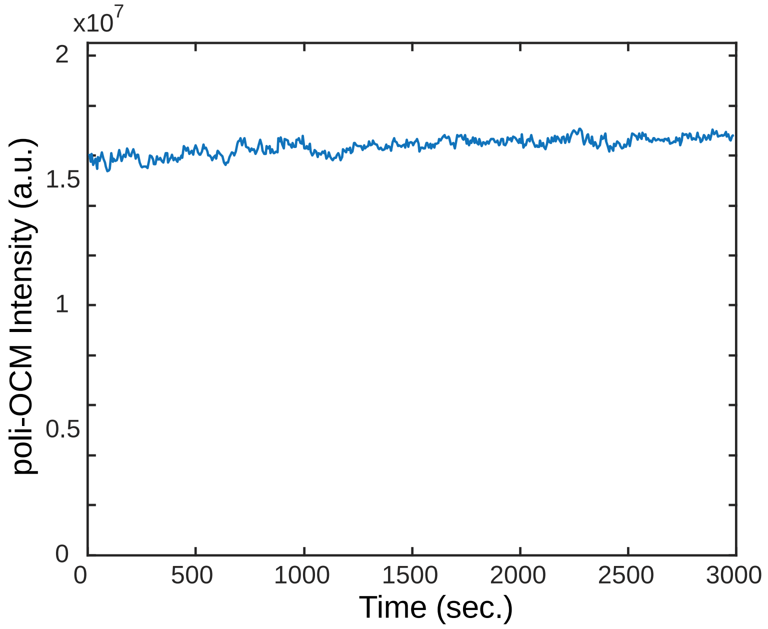

Figure S6: Integrated poli-OCM signal in the whole imaging volume over the imaging period. 500 3D poli-OCM tomograms were acquired continuously at a rate of 1/6Hz.

S.4. Image processing & analysis algorithm

The poli-OCM, as we have briefly described in S3 of the Supporting Information, is a Spectral Domain OCM system. Its underlying interferometric principle enables 3D volumetric imaging with only *x-y* scanning.[1-4] Figure S7 summarizes the algorithm we used to generate 3D tomograms. We subtracted a measured background spectrum from the interference spectrum

acquired at every position on the *x*-*y* plane. We then applied "k-mapping" which converts our spectra that is linear with wavelength λ to spectra linear in wavenumber k. We then obtain the 3D tomogram after a Fast Fourier Transform of these spectra. This procedure is repeated for all acquisition in the time series resulting in a 3D time-lapse data set.

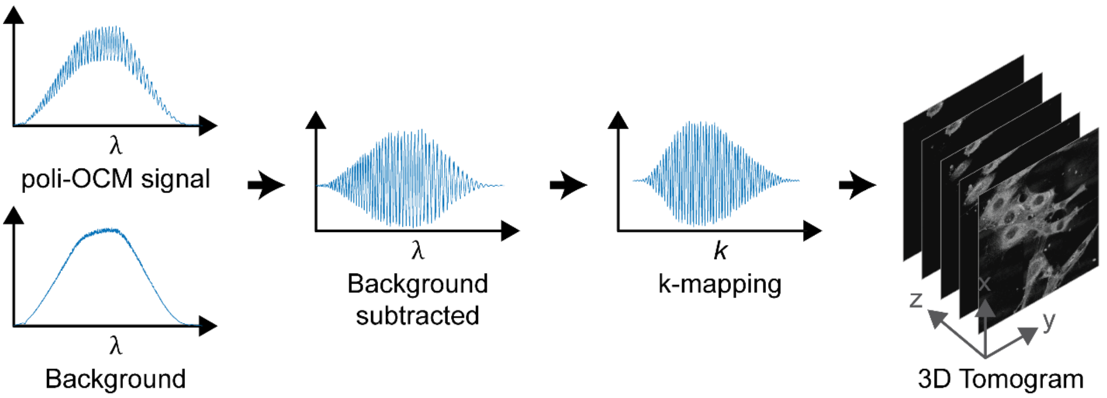

Figure S7: Summary of 3D tomogram calculation.

We then calculate the temporal autocorrelation of our poli-OCM signal following Equation S2.[5-7]

$$G_V(\tau) = \frac{\langle \delta i_V(t)\delta i_V(t+\tau)\rangle}{\langle i_V(t)\rangle\langle i_V(t+\tau)\rangle} = \frac{(T-\tau)\sum_{t=0}^{T-\tau}\delta i_V(t)\delta i_V(t+\tau)}{\sum_{t=0}^{T-\tau} i_V(t)\sum_{\tau}^{T} i_V(t)} \quad \text{(S2)}$$

Here $i_V(t)$ is the time series of intensity values at a given voxel, and T is the period or total length of the time series. The temporal intensity fluctuation is defined as $\delta i_V(t) = i_V(t) - \langle i_V(t)\rangle$ like in the main text. We then average the autocorrelation over a volume of 2×2×2 voxels (0.58µm×0.58µm×2.05µm) then fit the diffusion model using non-linear least squares solver; specifically we use the `lsqcurvefit` function of Matlab R2015b. Averaging over this sub-volume decreases the number of voxels our diffusion parameter maps (i.e. an initial 40×40×20 voxel space will be reduced to 20×20×10). To reduce computation time, we generate a mask using the dfOCM image of the cell to exclude voxels outside the cell volume. For the last part of our analysis, we only consider sub-volumes with autocorrelations fitted with an $R^2 \geq 0.85$. In addition, we also excluded sub-volumes having autocorrelations similar to sub-volumes outside the cell. Diffusion parameters were extracted only from the remaining sub-

volumes. Regions inside the cell that were excluded correspond to areas without AuNP labeled mitochondria.

Scanning, resolution, and sub-volume size: With the poli-OCM we are only limited by a maximum scan range for a given scanning speed/rate and pixel dimension. For example, a 512 x 512 pixel scan at 20 kHz A-Scan rate can only scan over 300x300 µm2 before we encounter instability with the galvanometric scanner. In theory, the opposite of higher pixel-to-micrometer ratio can be achieved by scanning a shorter range with a larger pixel dimension. However, we chose to oversample our point spread function (psf) but still limit our minimum region-of-interest/sub-volume to the size of our psf. Furthermore, switching to the visible spectrum would, in principle, improve the resolution but this would be a full redesign of the instrument.

S.5. Diffusion parameter maps

In addition to quantifying mitochondrial dynamics with the diffusion time, we extract the diffusion constant D and $\frac{1}{G(0)}$. Similar to Figure 4, we show the probability distribution and a rendered 3D map of these physical parameters in Figure S8. We calculate the diffusion constant D following

$$D = \frac{{r_0}^2}{4\tau_D} \tag{S3}$$

where τ_D is the extracted diffusion time an r_0 is the same as in Table S1. We divide both $pdf(D)$ and $pdf\left(\frac{1}{G(0)}\right)$ into 5 color-coded segments each corresponding to 20% of the cell volume and use these to generate the 3D maps.

The diffusion coefficient can be further related to viscosity by the Stokes-Einstein relation given by

$$D = \frac{k_B T}{6\pi\eta r_h}. \tag{S4}$$

k_B is the Boltzmann constant, T the temperature, η is the viscosity, and r_h is the hydrodynamic radius of the diffusing particle.

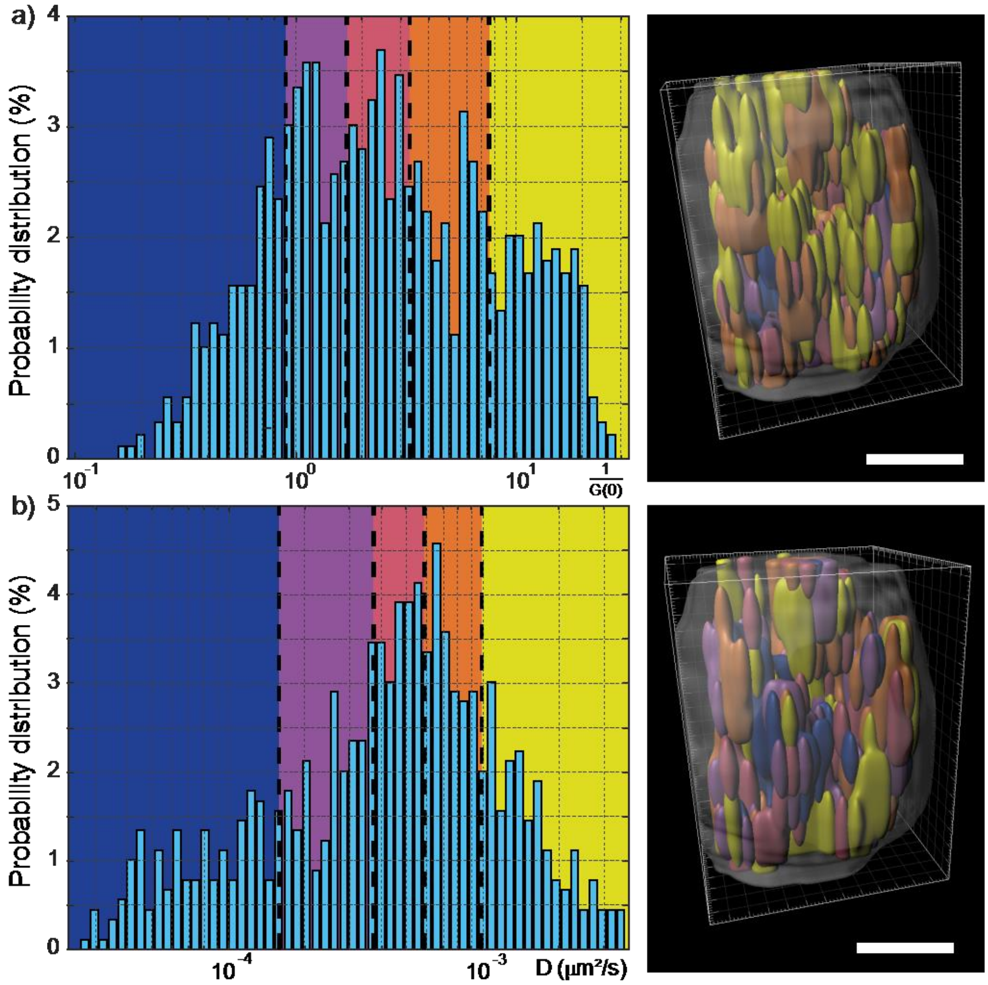

Figure S8: Probability distribution and rendered 3D map of a) mean number of AuNP labeled mitochondria and b) diffusion constant per sub-volume. Scalebar: 5µm

S.6. Details on diffusion time map

We separate the diffusion time map in Figure 4c and Figure 5a into the 5 intervals (Figure S9 b-f) of the probability distribution in Figure 5b. Furthermore, we include a 6th region, shown in white (Figure S9 a-f), which represents the volume within the cell where we extract no diffusion parameters. In this volume, we do not measure any poli-OCM signal and consider it as a region of "infinite" diffusion time. In addition, we observe that this volume has a good correlation with the location in the dfOCM images with lower scattering signal (darker or lower contrast). We expect the nucleus of the cell to be located within this volume.

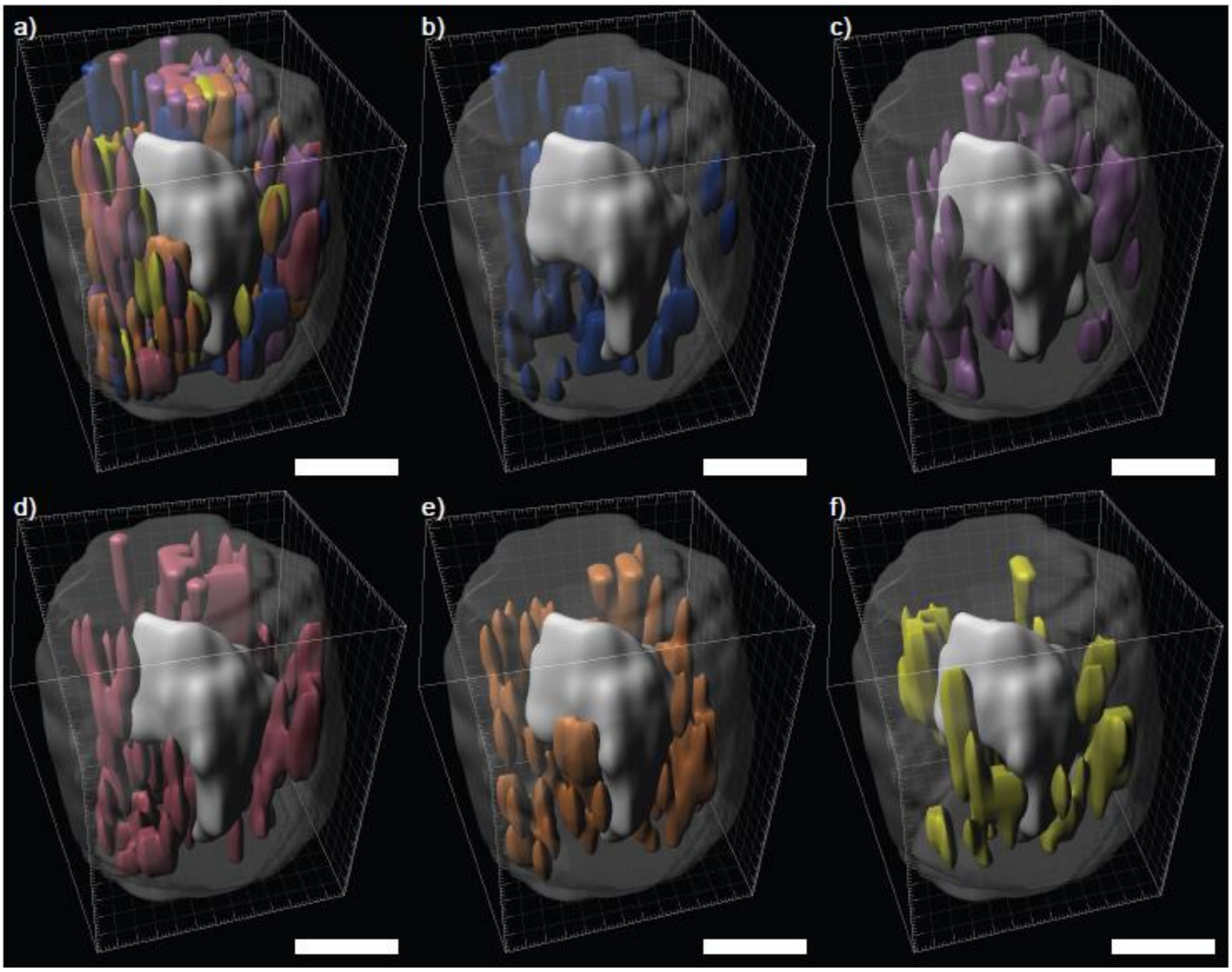

Figure S9: 3D rendering of AuNP labeled mitochondria diffusion time for each of the different cell segments (see main text Figure 4) including a volume with infinite diffusion time supposedly corresponding to the nucleus. Scalebar: 5µm

Supporting Videos

S.7. poli-OCM time lapse videos

We include a video of the 3D time lapse poli-OCM imaging. The video frames are maximum intensity projections with color-codes indicating the position of the AuNP labeled mitochondria in depth.

MOVIE S1: XY plane

MOVIE S2: XZ plane

MOVIE S3: YZ plane

References

1 Fercher, A. F., Drexler, W., Hitzenberger, C. K. & Lasser, T. Optical coherence tomography - principles and applications. *Rep Prog Phys* **66**, 239-303, doi:Pii S0034-4885(03)18703-9,Doi 10.1088/0034-4885/66/2/204 (2003).
2 Leitgeb, R., Hitzenberger, C. K. & Fercher, A. F. Performance of fourier domain vs. time domain optical coherence tomography. *Opt Express* **11**, 889-894, doi:10.1364/OE.11.000889 (2003).
3 Leitgeb, R. A., Villiger, M., Bachmann, A. H., Steinmann, L. & Lasser, T. Extended focus depth for Fourier domain optical coherence microscopy. *Opt Lett* **31**, 2450-2452, doi:Doi 10.1364/Ol.31.002450 (2006).
4 Villiger, M., Pache, C. & Lasser, T. Dark-field optical coherence microscopy. *Opt Lett* **35**, 3489-3491 (2010).
5 Broillet, S. *et al.* Optical coherence correlation spectroscopy (OCCS). *Opt Express* **22**, 782-802, doi:10.1364/OE.22.000782 (2014).
6 Octeau, V. *et al.* Photothermal Absorption Correlation Spectroscopy. *Acs Nano* **3**, 345-350, doi:10.1021/nn800771m (2009).
7 Paulo, P. M. R. *et al.* Photothermal Correlation Spectroscopy of Gold Nanoparticles in Solution. *J Phys Chem C* **113**, 11451-11457, doi:10.1021/jp806875s (2009).
